# From Injury to Impact: Optimizing Return-to-Play Outcomes and Reinjury Prevention via Four-Pillar Rehabilitation Strategy in Elite Football—A Clinical Study in a Sports Scenario

**DOI:** 10.3390/muscles5010011

**Published:** 2026-02-02

**Authors:** Ioannis Stathas, Nikos Koundourakis, Charalampos Christoforidis, George Kouvidis, Anna Christakou

**Affiliations:** 1 OFI Crete FC, 71303 Heraklion, Crete, Greece; nkoundourakis@oficretefc.com (N.K.); u2849833519@gmail.com (C.C.); kouvidisgeo@yahoo.com (G.K.); 2 Faculty of Sports Science and Physical Education, Metropolitan College, 71202 Heraklion, Crete, Greece; 3 Orthopedic Clinic, University General Hospital of Heraklion, 71500 Heraklion, Crete, Greece; 4 Laboratory of Biomechanics, Department of Physiotherapy, University of Peloponnese, 23100 Sparta, Greece; a.christakou@go.uop.gr

**Keywords:** prevention, on-field rehabilitation, return to play

## Abstract

Objectives: This clinical commentary presents a four-pillar rehabilitation framework implemented in the elite football setting of OFI Crete FC and designed to facilitate the return of football players to training and competitive play. The framework is structured around five core components: (a) effective load management during training and matches, (b) individualized rehabilitation programs and injury prevention strategies integrated within the recovery phase, (c) a novel on-field rehabilitation framework, and (d) an extended secondary prevention plan. Methods: This comprehensive approach was implemented over a three-year period with the OFI Crete FC football team and involved 87 elite professional players between the ages of 17 and 35. Throughout this time, 180 injuries were documented, ranging from mild to severe injuries. Results: The outcome illustrated that only 40% of these injuries led to players missing official matches, while the recurrence or follow-up injury rate was limited to just 10%. Over the course of the three years, a steady 60% decline in injury rates was observed. Conclusions: These findings emphasize the crucial importance of training load management, the integration of injury prevention strategies throughout the rehabilitation process, and the early initiation of on-field rehabilitation. Within the clinical setting of OFI Crete FC, the implementation of this integrated rehabilitation framework was associated with favorable observations in injury incidence, player absence days, and return-to-play timelines, which may reflect that the approach has potential benefits while remaining observational in nature.

## 1. Introduction

Today, in elite football, a key element affecting a player’s capacity to perform in games is a safe and timely return to the sports field after injury. Beyond the immediate absence from training and competition, injuries have a direct negative effect on players’ individual performance following their return to play. For example, Rossi et al. [1] analyzed external load and match participation data in elite football players before and after injury and reported that athletes who sustained moderate or severe injuries exhibited a significant reduction in match-starting appearances and maximal sprint speed after returning to competition. These impairments in individual performance may subsequently affect team performance across the competitive season. Indeed, injuries and the associated number of days players are absent from team activities have been shown to significantly influence league performance, with teams sustaining more injuries and experiencing longer absence durations being less likely to achieve their competitive objectives [2]. Furthermore, player absences due to injuries impose a substantial financial burden on professional clubs. Notably, a study conducted among La Liga teams [3] reported that the annual cost of muscle injuries amounts to approximately five million euros per team per season, reflecting broader financial losses related to reduced sporting success and associated revenues.

Moreover, a systematic review of professional football players from top-tier European leagues reported an injury incidence of 8.1 per 1000 h of exposure. Similarly, findings from an 11-year study on Union of European Football Associations (UEFA) Champions League teams revealed a comparably high injury rate of 7.7 injuries per 1000 h of exposure [4]. Also, recently, a three-year prospective study on elite youth soccer players reported that muscle injuries represented the most frequent type of injury, with an incidence rate of 11.0 injuries per 1000 h of exposure, the highest among all injury categories [5]. Specifically, regarding muscle injuries in professional elite football [6], a total of 2908 cases were recorded, corresponding to approximately 0.6 muscle injuries per player per season. Muscle injuries accounted for about 31% of all reported injuries and contributed to approximately 27% of the total number of days lost. Hamstring injuries are among the most frequent and recurrent injuries in football. The incidence of acute hamstring injury in soccer has been reported to range from 0.3 to 1.9 per 1000 h of exposure, depending on sex, competition level, and study population [7]. In addition, in a six-year prospective study of Major League Soccer (MLS) players, which includes professional teams participating in the top-tier football league of the United States and Canada, the overall injury incidence rate was reported to be 8.7 injuries per 1000 h of exposure, with the match injury incidence being 4.1 times higher than during training sessions (14.0 vs. 3.4 injuries per 1000 h, respectively) [8]. In women’s elite football, the overall injury incidence is notably high, with 6.7 injuries per 1000 h of exposure. The match injury incidence during the in-season was approximately four times higher than the training injury incidence during the in-season, a trend consistent with findings from previous UEFA injury studies. Thigh muscle injuries were the most frequent, accounting for 23% of all reported injuries, with hamstring injuries representing 12% and quadriceps injuries 11% of the total [9].

Modern rehabilitation frameworks in sports medicine emphasize individualized, criteria-based progression rather than rigid time-based protocols, guiding athletes from initial recovery through safe return to sport with clearly defined functional milestones. The 2016 Bern Consensus Statement [10] conceptualized return to sport as a multidimensional and collaborative risk management process, in which clinical, functional, psychological, and sport-specific factors are jointly considered along a continuum of return to participation, return to sport, and return to performance. Complementarily, the more recent evidence-informed recommendations for individualized rehabilitation and running progressions highlight the importance of graded loading, measurable load tolerance (e.g., strength, symmetry, movement control), and psychological readiness before full reintegration. Together, these frameworks establish a generalizable foundation for rehabilitation protocols across various musculoskeletal injuries, providing operational hypotheses and measurable indicators such as time to return, recurrence rates, and changes in injury burden [11].

Based on the importance of reducing injuries and shortening the time to return to play, over the past three years at OFI Crete FC, we have implemented a rehabilitation strategy based on four key principles: (a) clinical assessment and daily load management, (b) individualized rehabilitation protocols combined with injury prevention strategies integrated within the recovery process, (c) a novel framework for on-field rehabilitation, and (d) an expanded plan for secondary prevention. Grounded in OFI Crete FC’s four-pillar rehabilitation strategy, this study endeavors to elucidate the observational findings of the implemented protocol with respect to reducing the total incidence of injuries, minimizing the duration required for players to achieve full reintegration into training and competition, and decreasing the rate of injury recurrence. 

This clinical commentary presents the implementation of a four-pillar rehabilitation framework in an elite football setting, providing descriptive observations on injury trends, player reintegration, and practical clinical considerations for musculoskeletal recovery and adaptation. The focus is on sharing clinical insights and applied strategies rather than inferring causal relationships or evaluating protocol outcomes. The framework combines individualized load management, on-field functional reintegration, and extended secondary prevention within routine medical and performance practice. It highlights how these components are applied in daily rehabilitation workflows, emphasizing tissue adaptation, functional readiness, and the interaction between muscular and non-muscular systems during progression toward full return to training and competition. Its primary contribution lies in clarifying, illustrating, and effectively communicating professional procedures, serving to systematically formalize and coherently convey expert practices rather than presenting a wholly novel framework.

## 2. Materials and Methods

### 2.1. Design

This study was based on a retrospective observational approach aimed at analyzing data related to injuries, rehabilitation protocols, and return-to-play timelines. The data were collected over a continuous three-year period, from July 2022 to June 2025, within the professional football environment of OFI Crete FC. The study participants provided a written declaration of consent, and the study was approved by the Ethics Committee of the School of Health Studies of the University of Peloponnese (22622/17 September 2025).

### 2.2. Participants

Participants provided written, informed consent, and the clinical commentary was in agreement with the ethical principles of the Declaration of Helsinki A total of 87 injury cases were recorded among male professional football players aged between 18 and 35 years, with an accumulated 944 h of training and participation in 141 official matches, and with a total game time of approximately 212 h. The study assesses musculoskeletal injuries of the lower limb in male professional football players. These injuries include musculotendinous injuries and joint injuries, according to the Fuller et al., 2006, classification [12]. Specifically, muscle injuries accounted for approximately 60% of all recorded cases, representing the most frequent type of injury observed among participants. These included both acute and overuse musculotendinous lesions, which predominantly affect the hamstrings, quadriceps, and adductor muscle groups and are commonly reported in professional football players due to the high demands of sprinting, acceleration, and change-of-direction activities. The inclusion criteria were (a) musculoskeletal injuries sustained during the study period, (b) new injuries, and (c) secondary or recurrent injuries related to a previous musculoskeletal condition. The exclusion criteria included (a) non-musculoskeletal injuries, such as illness or concussion, and (b) cases with incomplete data concerning injury diagnosis, rehabilitation processes, or return-to-play outcomes. The injuries were categorized into mild (<7 days) and moderate and severe injuries (>7 days) [13]. This categorization allowed descriptive grouping of injuries for clinical interpretation and monitoring purposes. The injury incidence and injury burden were calculated based on the methodology outlined in the UEFA study [1,6,14].

### 2.3. Measurements and Procedure

The assessment of each injury was carried out by the team doctor, based on the UEFA study from Ekstrand et al. [15], and clinical tests for the lower limbs were carried out based on [16,17,18,19,20]. Where deemed necessary, imaging examinations were also performed. Throughout the process, all injured players followed a unified rehabilitation protocol, which was designed and implemented by the club’s medical and performance staff. It is worth noting that the performance staff remained consistent throughout the three-year period, providing continuity in the implementation of procedures and enhancing the reliability of the intervention outcomes.

Data collection was systematically conducted by the club’s medical and performance staff, following standardized internal documentation protocols. The collected data included detailed injury records, individualized rehabilitation plans, monitoring reports of external and internal loads, results from physical tests, and assessments of players’ readiness for full return to training and competitive play. All data were analyzed at the end of each competitive season, aiming to identify trends and evaluate the effectiveness of the implemented rehabilitation strategy.

The rehabilitation approach applied was individualized, tailored to the needs of each football player, taking into consideration the type of injury and history of previous injuries. The implementation process involved continuous and close collaboration among physicians, physiotherapists, performance coaches, and the technical staff. Decisions regarding return to play were based on objective data, performance assessments, and clearly defined functional criteria.

The football players’ progress was closely monitored through daily and weekly interdisciplinary meetings, during which the rehabilitation process was evaluated, and, when necessary, adjustments were made to the treatment plans or reintegration procedures.

The rehabilitation approach adopted in the present clinical commentary was based on a holistic and athlete-centered philosophy, aiming not only for safe and effective recovery but also for enhancement of the football players’ long-term resilience. This strategy was structured around four key pillars, which guided each stage of the return-to-play process. These pillars are presented in detail below.

The calculation of injury incidence, injury burden, and days of absence followed the standardized methodology established by the UEFA Elite Club Injury Study [1,6,14]. Specifically, injury incidence (II) was defined as the number of injuries per 1000 h of player exposure, including both training and match play, and was calculated as follows:

*II* = Number of injuries/Total exposure hours × 1000

Injury burden (IB) represents the total number of absence days per 1000 h of exposure, according to the formula

*IB* = Total number of absence days/Total exposure hours × 1000

Finally, days of absence (DA) were determined as the number of days between the date of injury and the date of full return to team training and competitive participation, expressed as

DA = Date of full return to team training and match participation − Date of injury

These definitions and computational formulas are internationally accepted and widely applied in sports injury epidemiology, ensuring consistency with prior large-scale investigations in professional football. Adhering to this methodological framework enables reliable comparison of injury incidence, burden, and recurrence trends across different seasons and studies, supporting the validity of the present analysis.

### 2.4. Four-Pillar Rehabilitation Strategy

I. Clinical assessment and daily load management

The daily load represents the total amount of physiological and mechanical stress an athlete experiences within a single day as a result of training and match participation. It integrates both external load (the objectively measured physical work, such as distance covered, accelerations, or session duration) and internal load (the athlete’s physiological and perceived response, such as heart rate or session-RPE). Monitoring daily load enables practitioners to balance training and recovery, minimize injury risk, and optimize performance outcomes by managing fluctuations in workload exposure [21]. Managing training load, the amount of stress placed on an athlete during training and competition, is extremely important during both training sessions and matches. By conducting regular evaluations to quantify workload and adjust training demands accordingly, the risk of overload and injury aggravation can be mitigated. This approach promotes consistent training participation and aligns with evidence-based injury prevention principles [21]. Daily clinical assessment of the football player also plays a crucial role in injury prevention, and effective load management requires continuous monitoring and adjustment based on the athlete’s clinical status—not solely on quantitative measurements [22]. At OFI Crete FC, a club competing in the Greek Super League, when a player sustained a minor injury (mild for <7 days), the medical team would assess the player and, based on clinical evaluation and in collaboration with the performance coach and technical staff, the player would train accordingly—either individually or with the team—depending on the stage of the injury, while managing training intensity and volume. For more significant injuries (moderate or severe >7 days), the player would stop training with the team and follow the appropriate rehabilitation protocol.

II. Tailored rehabilitation programs integrating preventive approaches

Primary prevention programs (team-based and individualized) and secondary prevention programs throughout the season are critical in reducing injury incidence during competition periods [23]. In our approach, we implemented primary injury prevention programs at the team level twice per week, and individualized programs three to four times per week, tailored to each football player’s deficits, identified during the pre-season screening. Furthermore, for every injury, whether moderate or severe (>7 days), we incorporated preventive exercises during the rehabilitation process to reduce the likelihood of subsequent injuries to the same or contralateral limb. As highlighted in a study on track and field athletes [24], athletes lost 50% more training and competition time due to recurrent injuries compared to their initial injuries. The preventive exercises were selected based on the type of injury. For example, proprioceptive exercises for the knee were used following quadriceps strains, and stretching exercises were included to preserve muscle flexibility in targeted muscle groups.

III. The On FI.RE Framework

On-field functional rehabilitation is extremely important for the safe and timely return of football players to training and competition. According to the ecological theory in sports, the earlier rehabilitation begins on the football pitch—the athlete’s natural environment where daily interactions occur—the faster they adapt to the demands of training and matches alongside their teammates. In the present study, we applied the On FI.RE. Framework (On Field rehabilitation Framework), which offers a structured, evidence-based approach for the safe reintegration of athletes into full training and competitive play. This framework emphasizes functional readiness and risk reduction, in alignment with contemporary return-to-play (RTP) decision-making guidelines. Additionally, it reduces the athlete’s average return-to-play time by approximately 7 days [25]. A key component of the On FI.RE. protocol is the systematic use of clinical criteria—including pain, range of motion (ROM), and swelling (edema)—to determine the appropriate timing for progression. Pain levels are continuously monitored through standardized scales (e.g., VAS), and athletes advance only when discomfort remains below predefined thresholds during and after functional tasks. Similarly, restoration of full or near-full ROM and the absence of joint effusion are prerequisites for initiating and intensifying on-field therapeutic exercise. These objective indicators allow for an earlier yet safe transition to field-based rehabilitation, ensuring that load tolerance, neuromuscular control, and functional adaptation develop in the athlete’s natural sporting environment while minimizing the risk of recurrence or delayed healing.

IV. Extended Secondary Prevention Phase

A structured and systematic injury prevention program is implemented for six months following the athlete’s return to play (RTP), depending on the severity of the initial injury. This program includes exercise-based interventions and individualized load management, in collaboration with the performance coach. Secondary prevention is a critical component of the rehabilitation process, as it has been reported that reinjuries account for up to 30% of all injuries in professional football [1]. Furthermore, studies show that recurrent injuries are associated with prolonged absence from competition and significant negative impacts on the athlete’s performance and career development [26,27]. These studies highlight the importance of secondary prevention after injury. At OFI Crete FC, we adhere to the fundamental principles of injury prevention as outlined in the literature [28,29,30]. Specifically, for the first two months after RTP, the individualized prevention program was conducted three times per week, followed by two sessions per week over the subsequent four months. The exercises were specific to the initial injury and focused on maintaining and enhancing muscular strength, improving range of motion (ROM), and ensuring optimal neuromuscular control to reduce reinjury risk and support sustained athletic performance throughout the competitive season.

## 3. Results

The observational findings showed that during this three-year period, moderate and severe injuries decreased by 48%. While mild injuries decreased by 35% in the second year, in the year the team participated in the cup final—which involved heightened demands associated with the goal of winning the cup—mild injuries rose by 50%. Regarding recurrences and subsequent injuries, during the 2022–2023 season, moderate and severe injury recurrences accounted for 20% of the total injuries; this percentage decreased to 19% in the 2024–2025 season and represented 5% of the total injuries. Recurrences represented 13% of mild injuries in 2022–2023 but dropped to less than 1% in 2024–2025.

Injury incidence, defined as the number of injuries resulting in missed training sessions or matches, was recorded as follows: during the 2022–2023 season, there were 2.2 injuries per 1000 h of training exposure and 28.1 injuries per 1000 h of match exposure. In 2023–2024, these rates declined to 0.74 injuries per 1000 h for training and 11.1 for matches. For the 2024–2025 season, the incidence slightly increased to 1.0 injury per 1000 h of training exposure and 11.5 injuries per 1000 h of match exposure. Muscle injuries represented the majority of all recorded cases, accounting for approximately 60% of the total injuries documented throughout the three-year observation period. These included both acute and overuse muscle lesions primarily affecting the hamstrings, quadriceps, and adductors. The overall injury incidence across all injury types during the data collection period was calculated to be 0.8 injuries per 1000 h of total exposure (training and match play combined), indicating a relatively low frequency compared to the rates typically reported in elite professional football environments.

Additionally, total player absence days decreased significantly from 1162 days in 2022–2023 to 468 days in 2024–2025. Correspondingly, the injury burden dropped from 100 lost days per 1000 h of training exposure and 476 per 1000 h of match exposure in 2022–2023, to 29.5 and 222 lost days per 1000 h, respectively, in 2024–2025.

Reinjury incidence followed a similar trend: in 2022–2023, the reinjury rate was 0.69 per 1000 h during training and 4.32 per 1000 h during matches. These figures declined to 0 and 1 per 1000 h, respectively, in subsequent seasons. All relevant data are presented in Table 1 and Table 2.

## 4. Discussion

### 4.1. Overview of the Four-Pillar Rehabilitation Strategy

The four-pillar rehabilitation strategy offers a clinically applicable and performance-aligned model that balances injury risk management with the physical demands of elite football. Its implementation at OFI Crete FC over a period of three competitive seasons allowed consistent application of the clinical framework.

### 4.2. Comparison with UEFA Benchmarks

Based on data from the UEFA Elite Club Injury Study [15], which outlined typical injury incidence, injury burden, and reinjury rates among professional football players, our observational findings indicate a substantial reduction in all three metrics over the past three seasons—placing OFI Crete FC below the average of Europe’s elite clubs. While the average injury incidence across European professional teams is approximately 3.4 injuries per 1000 h of training exposure and 23.8 injuries per 1000 h of match exposure, OFI Crete FC recorded significantly lower rates during the 2024–2025 season—showing a 70.6% reduction in training-related injuries and a 49.6% reduction in match-related injuries. It should be noted that while several studies are referenced in this manuscript to provide context on injury incidence, burden, and reinjury trends in elite football [1,6,14,15] none of these studies provide direct data from the UEFA regarding the seasons observed in this report. As a result, direct comparisons with UEFA injury rates are limited. The trends observed in our cohort should therefore be interpreted descriptively, and may not necessarily reflect current injury patterns across all elite European football clubs.

In terms of injury burden, European averages are reported to amount to 60.5 days lost per 1000 training hours and 504 days lost per 1000 match hours. At OFI Crete FC, the corresponding figures show decreases of approximately 51.2% and 56%, respectively.

Based on the observational findings, regarding reinjury incidence during the 2024–2025 season, OFI Crete FC demonstrated a 99% reduction compared to the European average during training and a 56.8% reduction during matches. These consistent improvements are largely attributed to the club’s structured rehabilitation protocols, built around four key pillars: load management, injury prevention, early intervention, and the On FI.RE. Framework.

### 4.3. Organizational Stability and Interdisciplinary Collaboration

The three-year uninterrupted application of this integrated model, combined with stable and interdisciplinary medical and performance staff, strengthens the internal validity of the findings. A consistent medical team minimizes procedural variability and facilitates adherence to uniform rehabilitation protocols. The literature has shown that such continuity in care, when combined with evidence-based practice, significantly reduces injury incidence and improves return-to-play (RTP) outcomes in elite football settings [31].

### 4.4. Load Management and Performance Integration

Central to the model is the integration of daily clinical assessment and individualized load management. Managing training volume and intensity allows for precise injury risk modulation and real-time training adaptations. Tailored management of mild injuries enables players to participate in modified or full training depending on readiness, maintaining rhythm and reducing detraining. This continuous engagement supports team performance continuity and tactical consistency [32].

### 4.5. Theoretical Framework: Ecological Dynamics and On-Field Rehabilitation

A distinctive component of the model is the early and progressive on-field rehabilitation, grounded in ecological dynamics theory. Transitioning from clinical to field-based work within an environment familiar to the athlete restores sport-specific neuromuscular control and facilitates psychosocial readiness for RTP.

The On FI.RE. Framework offers structured progressions in movement complexity, load tolerance, and decision-making under stress—key elements for safe and effective return to play [23]. By simulating realistic football-specific conditions, it bridges the gap between the clinic and field, ensuring that recovery is functionally complete rather than symptom-based.

### 4.6. Secondary Prevention and Long-Term Maintenance

Beyond the acute recovery phase, the strategy extends to a six-month secondary prevention phase post-RTP. This prolonged intervention includes individualized neuromuscular exercises, ongoing load regulation, and movement retraining to address residual deficits and reduce reinjury risk. All observations are presented descriptively, reflecting clinical experience rather than causal relationships. Evidence supports the long-term effectiveness of such programs in reducing recurrent injuries and improving athlete resilience in high-demand environments [28].

### 4.7. Interpretation of Reduced Injury Incidence

A lower injury incidence compared to elite football averages reflects an efficient system of monitoring, conditioning, and load regulation. When athletes sustain fewer injuries per 1000 h of exposure, it suggests that the balance between training stress and recovery is effectively managed. This outcome points toward an organizational structure emphasizing early risk detection, proactive intervention, and daily interdepartmental communication.

In environments where continuous data sharing occurs, even minor fluctuations in workload or fatigue can be corrected before developing into injuries—enhancing both individual and team performance continuity.

### 4.8. Analytical Comparison with Existing Literature

Several studies have emphasized the importance of individualized load management and monitoring strategies in reducing injury risk and recurrence. Ekstrand et al. [15] highlighted that inadequate load control and insufficient recovery are key contributors to both acute and overuse injuries [15], while Tabben et al. [33] demonstrated that systematic monitoring of internal and external load parameters can improve player availability [34].

Within this context, our findings align with existing evidence supporting integrated, criteria-based rehabilitation models that combine individualized load progression, ecological field exposure, and multidisciplinary decision-making to optimize RTP outcomes.

### 4.9. Function and Interrelation of the Four Pillars

The clinical observations described can be contextualized within the four-pillar rehabilitation model, in which each component plays a role in injury risk management and return-to-play planning.

Pillar 1: Individualized Load Management—Continuous monitoring of external (GPS-based) and internal (RPE) loads ensures players remain within an optimal physiological window. Clinical palpation complements quantitative data, allowing practitioners to detect localized tissue stress and adjust workloads accordingly [35].

Pillar 2: Tailored Rehabilitation and Preventive Approaches—Rehabilitation plans are adapted to each athlete’s injury profile and playing position. Preventive components (eccentric strength, proprioception, flexibility) are embedded early to enhance tissue resilience and correct asymmetries.

Pillar 3: On-Field Rehabilitation (On FI.RE. Framework)—This phase bridges the clinic and field, reintroducing sport-specific stimuli, decision-making, and reactive drills to ensure the player is both physically and cognitively ready for competition.

Pillar 4: Extended Secondary Prevention—Continuous post-return conditioning and periodic assessments maintain physical integrity, transforming rehabilitation into a long-term adaptive process.

### 4.10. Limitations and Future Directions

The limitations of this report include the absence of a control group, which precludes causal inference; the single-club context, which limits generalizability; and the lack of inferential statistical analysis. Future work in elite football settings may benefit from controlled or multi-club observational designs and the inclusion of biochemical, imaging, and neuromuscular assessments (e.g., CK, MRI, EMG) to further describe physiological adaptations related to return-to-play processes [36,37].

Additionally, the temporary rise in mild injuries during congested competition periods (e.g., cup finals) emphasizes the need for adaptive load regulation and psychological stress management during high-intensity phases.

Future research should aim to validate this framework in larger and more diverse athletic populations, employing controlled study designs and objective physiological, biomechanical, and imaging markers to better elucidate the mechanisms underlying recovery and adaptation. Overall, these results provide encouraging preliminary evidence supporting the potential value of holistic, athlete-centered rehabilitation frameworks in modern sports medicine.

### 4.11. Practical Implications

The findings of this study highlight the importance of close interdisciplinary collaboration among medical, physiotherapy, and performance departments in elite football. Effective communication and shared decision-making ensure individualized progression and continuity throughout rehabilitation.

The four-pillar model provides a practical framework that can be integrated into professional environments to support coordination and individualized return-to-play planning. The On FI.RE. Framework is particularly useful in bridging clinical recovery with sport-specific performance demands and can be adapted across different sports contexts.

Implementing such an athlete-centered rehabilitation system may significantly reduce injury incidence and recurrence, accelerate RTP timelines, and promote long-term player availability—ultimately improving both athlete welfare and sustained team success.

## 5. Descriptive Nature of the Observations

All observations reported in this manuscript are descriptive in nature and stem from routine clinical practice within a single elite football club. They represent the accumulated clinical experience of applying and managing the four-pillar rehabilitation framework in a real-world, high-performance environment, rather than measurements from an experimental, comparative, or inferential research design. Accordingly, these observations do not imply causal relationships between the rehabilitation framework and changes in injury incidence, return-to-play timelines, or reinjury rates. Instead, they offer contextual insights into how the framework was implemented, adapted, and integrated into daily clinical decision-making, with the goal of informing applied practice.

In conclusion, all reported patterns arise from direct clinical observation of the systematic use of the four-pillar rehabilitation protocol within an elite football setting. The documented injury patterns, return-to-play progression, and reintegration processes reflect experiential knowledge derived from daily practice. These observations should be interpreted as practice-based reflections on the operationalization of the framework in a real-world environment. They do not support causal claims, and any apparent associations between the protocol and observed patterns represent descriptive clinical observations rather than verified evidence of effectiveness.

## 6. Conclusions

The present clinical commentary describes the implementation of a structured, four-pillar rehabilitation strategy in elite football. By integrating individualized load management, injury prevention strategies, early on-field functional rehabilitation, and extended secondary prevention, the model provides a comprehensive and practically applicable framework for athlete care. Observations from the three-year application of this approach at OFI Crete FC indicate lower injury incidence, burden, and recurrence; however, these patterns should be interpreted descriptively, given the absence of statistical analyses and a control group.

This clinical commentary also highlights the importance of interdisciplinary collaboration and consistent communication between medical, physiotherapy, and performance staff in supporting rehabilitation processes and maintaining player participation. Several methodological limitations should be noted, including the relatively small sample size, the absence of a control or comparison group, and the single-club context, which may limit the generalizability of these descriptive observations.

## Figures and Tables

**Table 1:** Moderate and severe injury * data table for OFI Crete FC over the last three years.

Season	Number of Trainings	Number of Games	Number of Injuries		Total Hours of Exposure		Number of Reinjury or Subsequent Injury		Injury Incidence/1000 h of Exposure		Injury Burden/1000 h of Exposure		Reinjury Incidence/1000 h of Exposure	
			T **	G **	T	G	T	G	T	G	T	G	T	G
2022–2023	221	44	16	26	7182	924	5	4	2.2	28.1	100	476	0.69	4.32
2023–2024	247	47	6	11	8027	987	0	2	0.74	11.1	26.1	216	0	2.02
2024–2025	244	50	8	12	7925	1050	0	1	1	11.5	29.5	222	0	0.95

* Moderate and severe injury: moderate: 8–28 days of absence; severe: >28 days of absence. ** T = training; G = game.

**Table 2:** Mild * injury data table for OFI Crete FC over the last three years.

Season	Number of Trainings	Number of Games	Total Hours of Exposure	Number of Injuries	Number of Reinjury or Subsequent Injury	Games Missed
2022–2023	221	44	8850	38	5	0
2023–2024	247	47	7350	24	1	0
2024–2025	244	50	7350	47	1	0

* Mild: moderate injury with <7 days of absence.

## Data Availability

The dataset is not available because it contains sensitive team data.
